# Acute Histologic Chorioamnionitis Is a Risk Factor for Adverse Neonatal Outcome in Late Preterm Birth after Preterm Premature Rupture of Membranes

**DOI:** 10.1371/journal.pone.0079941

**Published:** 2013-12-04

**Authors:** Seung Mi Lee, Jeong Woo Park, Byoung Jae Kim, Chan-Wook Park, Joong Shin Park, Jong Kwan Jun, Bo Hyun Yoon

**Affiliations:** 1 Department of Obstetrics and Gynecology, Seoul National University College of Medicine, Seoul, Korea; 2 Department of Obstetrics and Gynecology, Seoul Metropolitan Government Seoul National University Boramae Medical Center, Seoul, Korea; 3 Department of Obstetrics and Gynecology, Inje University College of Medicine, Ilsan-Paik Hospital, Gyeonggi, Korea; Hôpital Robert Debré, France

## Abstract

**Background:**

The objective of this study was to determine whether acute histologic chorioamnionitis is associated with adverse neonatal outcomes in late preterm infants who were born after preterm PROM.

**Methodology/Principal Findings:**

The relationship between the presence of acute histologic chorioamnionitis and adverse neonatal outcome was examined in patients with preterm PROM who delivered singleton preterm newborns between 34 weeks and 36 6/7 weeks of gestation. Nonparametric statistics were used for data analysis. The frequency of acute histologic chorioamnionitis was 24% in patients with preterm PROM who delivered preterm newborns between 34 weeks and 36 6/7 weeks of gestation. Newborns born to mothers with histologic chorioamnionitis had significantly higher rates of adverse neonatal outcome (74% vs 51%; p<0.005) than those without histologic chorioamnionitis. This relationship remained significant after adjustment for gestational age at preterm PROM, gestational age at delivery, and exposure to antenatal corticosteroids.

**Conclusions/Significance:**

The presence of acute histologic chorioamnionitis is associated with adverse neonatal outcome in late preterm infants born to mothers with preterm PROM.

## Introduction

The rate of preterm deliveries rose steadily in recent years, from 9.4% in 1981 to 12.8% in 2006 according to the vital statistics in USA [1]–[3], and late preterm birth (34–36 6/7 weeks) represents 74% of all preterm deliveries [2]. Traditionally, little attention has been paid to late preterm neonates, regarded as “near-term” or “near-normal” neonates. However, recent studies have reported that late preterm birth is associated with neonatal morbidity and mortality [4]–[18], and hospital costs and health care utilization during the first year of life are also increased in these infants [9], [19], [20]. There is also increasing concern on long-term medical and behavioral morbidity, such as cerebral palsy, attention problems, antisocial behavior, and impaired cognitive and academic performances at school age [21]–[26]. With these evidences, Eunice Kennedy Shriver National Institute of Child Health and Human Development of the National Institutes of Human workshop stressed the importance of late preterm birth and called for increased research regarding the risk factors for short- and long-term morbidity for these late preterm infants [27].

Strong evidences support the relationship between intra-uterine inflammation and adverse neonatal morbidities in preterm birth, but most of these studies have focused on preterm birth <34 or 35 weeks' gestation [28]–[32]. At present, little is known about the antenatal conditions which increase the significant morbidity of late preterm neonates, and there is also a paucity of information regarding the relationship between intra-uterine inflammation and neonatal outcomes in late preterm infants.

To address this issue, we undertook this study to determine whether the presence of acute histologic chorioamnionitis is associated with the occurrence of adverse neonatal outcome in late preterm infants born to mothers with preterm premature rupture of membranes (PROM).

## Methods

### Study design

The study population consisted of consecutive patients with preterm PROM who delivered singleton live preterm newborns (gestational age between 34 weeks and 36 6/7 weeks) in Seoul National University Hospital between August 1998 and August 2009. Cases with major fetal anomalies were excluded from analysis. The relationship between the presence of acute histologic chorioamnionitis and the occurrence of adverse neonatal outcome was assessed.

We follow the ethical standards for human experimentation established in the Declaration of Helsinki. The Institutional Review Board of Seoul National University Hospital approved the collection and use of clinical samples and information for research purposes before the experiment was started. Participants provided their written consent to participate and written consent was obtained from the next of kin, caretakers, or guardians on the behalf of the minors/children participants involved. The Seoul National University has a Federal Wide Assurance (FWA) with the Office for Human Research Protections (OHRP) of the Department of Health and Human Services (DHHS) of the United States.

### Acute histologic chorioamnionitis

Tissue samples were obtained for histopathologic examination; tissue sections included the chorion-amnion, the chorionic plate, and the umbilical cord. These samples were fixed in 10% neutral buffered formalin and embedded in paraffin. Sections of tissue blocks were stained with hematoxylin and eosin. Acute histologic chorioamnionitis was defined as the presence of acute inflammatory changes in the extra-placental membranes or the chorionic plate of the placenta: the acute inflammation of amnion and chorion-decidua was defined as the presence of at least one focus of more than 5 neutrophils, and the acute inflammation of the chorionic plate was defined as the presence of more than one focus of at least 10 neutrophils; funisitis was diagnosed in the presence of neutrophil infiltration into the umbilical vessel walls or Wharton's jelly with the use of criteria that were published previously [33].

### Neonatal outcomes

Adverse neonatal outcome was defined as the presence of major morbidity (respiratory distress syndrome, intraventricular hemorrhage (≥grade II), necrotizing enterocolitis, proven or suspected congenital sepsis, early pneumonia, or bronchopulmonary dysplasia) and/or minor morbidity (seizure, treatment of apnea/bradycardia, need for respiratory assistant such as continuous positive airway pressure or ventilator, need for surfactant, reflux, hypoglycemia, longer duration to achieve full feeds, hyperbilirubinemia requiring phototherapy, blood transfusion for anemia, or longer admission to neonatal intensive care unit) (Table 1). Major neonatal morbidity was diagnosed according to the definitions previously described in detail [28]. The criterion for minor neonatal morbidity is described in Table 1, which was from the criteria of Bastek et al [10] with modification.

**Table 1:** Definition of adverse neonatal outcome.

Major morbidity	Minor morbidity	
Proven and/or suspected sepsis†	CNS:	Neonatal seizure
Respiratory distress syndrome		Treatment of apnea/bradycardia
Intraventricular hemorrhage (grades II–IV)	PULM:	Respiratory assistance (CPAP, ventilator)
Necrotizing enterocolitis		Need for surfactant
Pneumonia	GI:	Reflux
Bronchopulmonary dysplasia		Hypoglycemia (<50 mg/dl)
		Longer than 4 days to achieve full per os/nasogastric feeds
	HEME:	Hyperbilirubinemia requiring phototherapy (>3 days)
		Blood transfusion for anemia
	General:	Admission to the NICU ≥8 days

CNS: central nervous system; PULM: pulmonary; CPAP: continuous positive airway pressure; GI: gastrointestinal; HEME: hematology; NICU: neonatal intensive care unit.

† Proven neonatal sepsis: positive blood culture results (≤72 hours of delivery).

Suspected neonatal sepsis: presence of ≥2 of the following criteria, in the absence of a positive blood culture (≤72 hours of delivery); white blood cell count ≤5000 cells/mm^3^ or ≥24,000 cells/mm^3^, polymorphonuclear leukocyte count ≤1800 cells/mm^3^, ratio of band cells to total neutrophils ≥0.2, positive results of gastric aspiration for polymorphonuclear leukocytes showing ≥5 white blood cells per high power field, erythrocyte sedimentation rate ≥15 mm/min, positive result of C-reactive protein assay, platelet count ≤80,000 cells/mm^3^, or the presence of meningitis, urinary tract infection, or pneumonia as proven by culture.

### Statistical analysis

Proportions were compared with Fisher's exact test, and comparisons of continuous variables between groups were performed with a Mann-Whitney U test or Kruskal-Wallis analysis of variance as appropriate. Logistic regression analysis was conducted for multivariate analysis. The confounding variables in multiple logistic regression analysis were selected according to the analysis of univariate analysis as risk factors for adverse neonatal outcome (p<0.2). A p-value<0.05 was considered significant. A prior sample calculation was performed to determine how many patients would be needed to detect a 40% of increase in the frequency of adverse neonatal outcome in the presence of acute histologic chorioamnionitis. We estimated that the risk of adverse neonatal outcome would be 50% according to the report of Bastek et al [10]. Assuming 80% power, a type 1 error of 5%, and an estimated rate of 3∶7 between those with and without acute histologic chorioamnionitis (The frequency of acute histologic chorioamnionitis at term was reported as 23% [34] and that in preterm PROM before 35 weeks was reported as 50% [35] in our previous reports), we determined we would require 234 patients who delivered at late preterm.

## Results

### Study population

During the study period, 256 singleton pregnant women delivered late preterm infants (gestational age at delivery between 34 and 36 6/7 weeks) without major anomalies after preterm PROM. Among these women, the results of histopathologic examination of the placenta were not available in 12 patients. A total of 244 cases were included in this study.

### Clinical characteristics and pregnancy outcomes

Acute histologic chorioamnionitis and funisitis were diagnosed in 24% (58/244) and 10% (25/244) of cases, respectively. Table 2 describes the clinical characteristics and pregnancy outcomes of the study population according to the presence or absence of acute histologic chorioamnionitis. Group of patients with acute histologic chorioamnionitis had significantly lower gestational age at PPROM, lower gestational age at delivery, and had higher rate of corticosteroid use with marginal significance (p = 0.06). However, there was no significant difference in the median maternal age and proportions of nulliparity, history of preterm birth, use of tocolytics/antibiotics, clinical chorioamnionitis, and rate of cesarean delivery between the two groups.

**Table 2:** Characteristics and pregnancy outcomes of study population according to the presence or absence of acute histologic chorioamnionitis.

Characteristics	Histologic chorioamnionitis (−) (n = 186)	Histologic chorioamnionitis (+) (n = 58)	p
Maternal age*	31 (23–43)	31 (16–40)	0.80
Nulliparity	109 (59%)	32 (55%)	0.65
History of preterm birth	20 (11%)	6 (10%)	1.00
Gestational age at PPROM*	35.6 (21.7–36.9)	34.9 (27.7–36.7)	<0.005
Duration of ROM (days)*	0.70 (0.05–86.06)	1.73 (0.17–53.76)	<0.001
Antenatal corticosteroids	33 (18%)	17 (29%)	0.064
Tocolytics	18 (10%)	10 (17%)	0.15
Antenatal antibiotics	175 (94%)	57 (98%)	0.30
Clinical chorioamnionitis	1 (1%)	1 (2%)	0.42
Cesarean delivery	56 (30%)	18 (31%)	0.87
Gestational age at delivery*	35.7 (34.0–36.9)	35.3 (34–36.7)	<0.01

PPROM, preterm premature rupture of membranes; ROM, rupture of membranes.

* Median and range.

### Adverse neonatal outcome

The frequency of adverse neonatal outcome was 56% (137/244): 7% (18/244) with major morbidity and 55% (133/244) with minor morbidity. Table 3 compares the neonatal outcomes according to the presence or absence of acute histologic chorioamnionitis. Neonates born to mothers with acute histologic chorioamnionitis had higher frequency of major morbidity, minor morbidity, and adverse neonatal outcome than those without acute histologic chorioamnionitis, and this difference in the frequency of major morbidity and adverse neonatal outcome remained significant after adjustment for gestational age at delivery. Table 4 shows the result of logistic regression analysis for adverse neonatal outcomes. After adjustment for potential confounding variables (gestational age at PPROM, duration of ROM, the use of antenatal corticosteroids or tocolytics, cesarean delivery, and gestational age at delivery, all of which were associated with the risk of adverse neonatal outcome in univariate analysis), the presence of acute histologic chorioamnionitis was a significant risk factor for the subsequent development of adverse neonatal outcome.

**Table 3:** Neonatal outcome according to the presence or absence of acute histologic chorioamnionitis.

Characteristics	Histologic chorioamnionitis (−) (n = 186)	Histologic chorioamnionitis (+) (n = 58)	P (unadjusted)	p (adjusted)†
Birthweight*	2570 (1390–4320)	2445 (1690–4170)	<0.05	(−)
1-Min Apgar score <7	23 (12%)	11 (19%)	0.20	NS
5-Min Apgar score <7	3 (2%)	3 (5%)	0.15	NS
Duration of admission (days)*	7 (2–32)	8 (3–23)	<0.05	(−)
**Major morbidity**	7 (4%)	11 (19%)	<0.001	<0.001
Proven and/or suspected sepsis (proven sepsis)	4 (2%)	9 (16%)	<0.001	<0.001
	2 (1%)	5 (9%)	<0.01	<0.05
Respiratory distress syndrome	3 (2%)	2 (3%)	0.34	NS
Intraventricular hemorrhage (≥grades II)	0 (0%)	0 (0%)	(−)	(−)
Necrotizing enterocolitis	0 (0%)	0 (0%)	(−)	(−)
Pneumonia	0 (0%)	0 (0%)	(−)	(−)
Bronchopulmonary dysplasia	0 (0%)	0 (0%)	(−)	(−)
**Minor morbidity**	92 (50%)	41 (71%)	<0.01	0.063
Neonatal seizure	0 (0%)	0 (0%)	(−)	(−)
Treatment of apnea/bradycardia	3 (2%)	3 (5%)	0.15	NS
Respiratory assistance (CPAP, ventilator)	5 (3%)	2 (3%)	0.67	NS
Need for surfactant	2 (1%)	2 (3%)	0.24	NS
Reflux	2 (1%)	0 (0%)	1.00	NS
Hypoglycemia (<50 mg/dl)	30 (16%)	12 (21%)	0.43	NS
Longer than 4 days to achieve full per os/nasogastric feeds	24 (13%)	17 (29%)	<0.01	<0.05
Hyperbilirubinemia requiring phototherapy (>3 days)	56 (30%)	28 (48%)	<0.05	<0.05
Blood transfusion for anemia	1 (1%)	0 (0%)	1.00	NS
Admission to NICU ≥8 days	35 (19%)	16 (28%)	0.19	NS
**Adverse neonatal outcome** §	94 (51%)	43 (74%)	<0.005	<0.05

CPAP, continuous positive airway pressure; NICU, neonatal intensive care unit.

* Median and range.

† Adjustment for gestational age at PPROM, duration of ROM, the use of antenatal corticosteroid or tocolytics, cesarean delivery, and gestational age at delivery.

§ Adverse neonatal outcome was defined as the presence of major morbidity and/or minor morbidity.

**Table 4:** Relationship of significant variables in predicting adverse neonatal outcome in infants who were born at late preterm after preterm premature rupture of membranes by multiple logistic regression analysis with backward elimination.

Variables	Adjusted OR	95% CI	p value
Acute histologic chorioamnionitis	2.281	1.111–4.682	<0.05
Gestational age at delivery	0.470	0.314–0.702	<0.001
Antenatal corticosteroids	2.871	1.103–7.476	<0.05
Cesarean delivery	4.065	2.052–8.053	<0.001

Neonates born to mothers with funisitis had also higher frequency of major morbidity, minor morbidity, and adverse neonatal outcome than those without funisitis, but this difference did not reach statistical significance (major morbidity, 16% vs. 6%, p = 0.097; minor morbidity, 64% vs. 53%, p = 0.40, adverse neonatal outcome, 72% vs. 54%, p = 0.14).

## Discussion

### The principal findings of this study

1) The frequency of acute histologic chorioamnionitis was 24% in late preterm infants who were born after preterm PROM; 2) The presence of acute histologic chorioamnionitis was a risk factor for the subsequent development of adverse neonatal outcome even after adjustment for gestational age at delivery and exposure to antenatal corticosteroids.

### Histologic chorioamnionitis as a risk factor for adverse neonatal outcome

Modest but increasing interest has been raised on the increased neonatal morbidity in late preterm infants when compared to that in term infants, but the risk factors which increase the neonatal morbidity rate in these late preterm infants are still unknown. Previous studies showed that histologic chorioamnionitis/funisitis is associated with adverse neonatal morbidities in preterm birth at <34 or 35 weeks' gestation [28]–[32]. But, there is a paucity of information regarding the relationship between intra-uterine inflammation and adverse neonatal outcomes in late preterm infants.

Recently, Bastek et al [36] reported that preterm labor episode prior to 34 weeks was associated with adverse neonatal morbidities in late preterm neonates, and hypothesized that early preterm labor episode is a surrogate for intra-uterine inflammation and is responsible for adverse outcomes in these neonates. In the current study, we demonstrated that the presence of acute histologic chorioamnionitis is a risk factor for the occurrence of adverse neonatal outcome in late preterm infants delivered after preterm PROM. We believe this observation has important clinical and research implication, arousing attention to the importance of intra-uterine inflammation in late preterm.

### The effect of antenatal corticosteroid on neonatal outcome in late preterm

Whether late preterm infants would benefit from antenatal corticosteroid in terms of reducing neonatal respiratory or other morbidities is not well determined, although antenatal corticosteroid is proven to be associated with reduced risk of respiratory distress syndrome, intraventricular hemorrhage, necrotizing enterocolitis, and neonatal death in preterm birth at <34 weeks [37]–[39]. In the current study, use of antenatal corticosteroid was a risk factor for the occurrence of adverse neonatal outcome, even after adjustment for gestational age at delivery and acute histologic chorioamnionitis (Table 4). This is consistent with the result of Bastek et al., who also showed that infants who were exposed to antenatal corticosteroid due to preterm labor episode were at high risk for adverse neonatal outcome [36]. Other previous studies reported that antenatal corticosteroid did not reduce the incidence of respiratory disorders in late preterm neonates [40], [41]. However Ventolini et al reported that exposure to antenatal corticosteroids between 24 and 34 weeks was associated with a lower incidence of respiratory disorders in late preterm birth [42]. But their study population was not limited to late preterm birth following spontaneous birth, and it is possible that the indication of delivery might be the confounding factor for delivery outcome [36], [43], [44]. Further studies are needed to address whether antenatal corticosteroid improves outcomes of late preterm infants.

### The strength and weakness of the study

The strength of the current study is that the study population consisted of consecutive patients with late preterm birth after preterm PROM in one institution. During the study period, 259 singleton pregnant women delivered late preterm neonates without major anomalies after preterm PROM, and among these women, the results of histopathologic examination of the placenta were available in most cases (95%, 244/256). And our data only included patients who delivered after preterm PROM, consisting of homogenous group for the analysis between intra-uterine inflammation and adverse neonatal outcome.

### Unanswered questions and proposal for future research

There are several questions that need to be studied in further research. First, is the presence of acute histologic chorioamnionitis a risk factor for adverse neonatal outcome in late preterm infants who were born after preterm labor or medically indication? Delivery indication itself can affect the neonatal outcomes, and this also needs to be evaluated. Second, can antibiotic treatment to control intra-uterine infection and/or inflammation reduce adverse neonatal outcome in late preterm infants? Lastly, is the presence of acute histologic chorioamnionitis a risk factor for long-term neonatal morbidity such as cerebral palsy in late preterm infants? Although some neonatal outcomes which were evaluated in the current study have been known as risk factors for long-term morbidities [45]–[48], further studies may be needed to verify the relationship between intra-uterine inflammation and long-term outcomes of late preterm infants.

We believe that our data would help clinicians to counsel and predict the outcome of late preterm neonates. Furthermore, these data will compel active investigation into long term follow up of late preterm neonates to assess their risk for long term adverse outcome and determine interventions such as antibiotics will decrease neonatal morbidity.

In conclusion, the presence of acute histologic chorioamnionitis is an independent risk factor for the occurrence of adverse neonatal outcome in late preterm infants born to mothers with preterm PROM.

## References

[1] Martin JA, Hamilton BE, Sutton PD, Ventura SJ, Menacker F, et al (2006) Births: final data for 2004. Natl Vital Stat Rep 55: 1–101. 17051727

[2] Davidoff MJ, Dias T, Damus K, Russell R, Bettegowda VR, et al (2006) Changes in the gestational age distribution among U.S. singleton births: impact on rates of late preterm birth, 1992 to 2002. Semin Perinatol 30: 8–15. 16549207 10.1053/j.semperi.2006.01.009

[3] Martin JA, Hamilton BE, Ventura SJ, Osterman MJ, Kirmeyer S, et al (2011) Births: final data for 2009. Natl Vital Stat Rep 60: 1–70. 22670489

[4] Kramer MS, Demissie K, Yang H, Platt RW, Sauve R, et al (2000) The contribution of mild and moderate preterm birth to infant mortality. Fetal and Infant Health Study Group of the Canadian Perinatal Surveillance System. JAMA 284: 843–849. 10938173 10.1001/jama.284.7.843

[5] Seubert DE, Stetzer BP, Wolfe HM, Treadwell MC (1999) Delivery of the marginally preterm infant: what are the minor morbidities? Am J Obstet Gynecol 181: 1087–1091. 10561623 10.1016/s0002-9378(99)70086-4

[6] Arnon S, Dolfin T, Litmanovitz I, Regev R, Bauer S, et al (2001) Preterm labour at 34–36 weeks of gestation: should it be arrested? Paediatr Perinat Epidemiol 15: 252–256. 11489153 10.1046/j.1365-3016.2001.00357.x

[7] Sarici SU, Serdar MA, Korkmaz A, Erdem G, Oran O, et al (2004) Incidence, course, and prediction of hyperbilirubinemia in near-term and term newborns. Pediatrics 113: 775–780. 15060227 10.1542/peds.113.4.775

[8] Gilbert WM, Nesbitt TS, Danielsen B (2003) The cost of prematurity: quantification by gestational age and birth weight. Obstet Gynecol 102: 488–492. 12962929 10.1016/s0029-7844(03)00617-3

[9] Wang ML, Dorer DJ, Fleming MP, Catlin EA (2004) Clinical outcomes of near-term infants. Pediatrics 114: 372–376. 15286219 10.1542/peds.114.2.372

[10] Bastek JA, Sammel MD, Pare E, Srinivas SK, Posencheg MA, et al (2008) Adverse neonatal outcomes: examining the risks between preterm, late preterm, and term infants. Am J Obstet Gynecol 199: 367 e361–368. 18928976 10.1016/j.ajog.2008.08.002

[11] Khashu M, Narayanan M, Bhargava S, Osiovich H (2009) Perinatal outcomes associated with preterm birth at 33 to 36 weeks' gestation: a population-based cohort study. Pediatrics 123: 109–113. 19117868 10.1542/peds.2007-3743

[12] Colin AA, McEvoy C, Castile RG (2010) Respiratory morbidity and lung function in preterm infants of 32 to 36 weeks' gestational age. Pediatrics 126: 115–128. 20530073 10.1542/peds.2009-1381 PMC3000351

[13] McIntire DD, Leveno KJ (2008) Neonatal mortality and morbidity rates in late preterm births compared with births at term. Obstet Gynecol 111: 35–41. 18165390 10.1097/01.AOG.0000297311.33046.73

[14] Shapiro-Mendoza CK, Tomashek KM, Kotelchuck M, Barfield W, Nannini A, et al (2008) Effect of late-preterm birth and maternal medical conditions on newborn morbidity risk. Pediatrics 121: e223–232. 18245397 10.1542/peds.2006-3629

[15] Tomashek KM, Shapiro-Mendoza CK, Davidoff MJ, Petrini JR (2007) Differences in mortality between late-preterm and term singleton infants in the United States, 1995–2002. J Pediatr 151: 450–456, 456 e451. 17961684 10.1016/j.jpeds.2007.05.002

[16] Laughon SK, Reddy UM, Sun L, Zhang J (2010) Precursors for late preterm birth in singleton gestations. Obstet Gynecol 116: 1047–1055. 20966688 10.1097/AOG.0b013e3181f73f97 PMC3014049

[17] Celik IH, Demirel G, Canpolat FE, Dilmen U (2013) A common problem for neonatal intensive care units: late preterm infants, a prospective study with term controls in a large perinatal center. J Matern Fetal Neonatal Med 26: 459–462. 23106478 10.3109/14767058.2012.735994

[18] Mally PV, Hendricks-Munoz KD, Bailey S (2013) Incidence and etiology of late preterm admissions to the neonatal intensive care unit and its associated respiratory morbidities when compared to term infants. Am J Perinatol 30: 425–431. 23096053 10.1055/s-0032-1326989

[19] Bird TM, Bronstein JM, Hall RW, Lowery CL, Nugent R, et al (2010) Late preterm infants: birth outcomes and health care utilization in the first year. Pediatrics 126: e311–319. 20603259 10.1542/peds.2009-2869

[20] Berard A, Le Tiec M, De Vera MA (2012) Study of the costs and morbidities of late-preterm birth. Arch Dis Child Fetal Neonatal Ed 97: F329–334. 22933090 10.1136/fetalneonatal-2011-300969

[21] Nomura Y, Rajendran K, Brooks-Gunn J, Newcorn JH (2008) Roles of perinatal problems on adolescent antisocial behaviors among children born after 33 completed weeks: a prospective investigation. J Child Psychol Psychiatry 49: 1108–1117. 18673404 10.1111/j.1469-7610.2008.01939 PMC2585154

[22] Huddy CL, Johnson A, Hope PL (2001) Educational and behavioural problems in babies of 32–35 weeks gestation. Arch Dis Child Fetal Neonatal Ed 85: F23–28. 11420317 10.1136/fn.85.1.F23 PMC1721280

[23] Talge NM, Holzman C, Wang J, Lucia V, Gardiner J, et al (2010) Late-preterm birth and its association with cognitive and socioemotional outcomes at 6 years of age. Pediatrics 126: 1124–1131. 21098151 10.1542/peds.2010-1536

[24] Gray RF, Indurkhya A, McCormick MC (2004) Prevalence, stability, and predictors of clinically significant behavior problems in low birth weight children at 3, 5, and 8 years of age. Pediatrics 114: 736–743. 15342847 10.1542/peds.2003-1150-L

[25] Harijan P, Boyle EM (2012) Health outcomes in infancy and childhood of moderate and late preterm infants. Semin Fetal Neonatal Med 17: 159–162. 22417643 10.1016/j.siny.2012.02.002

[26] Lipkind HS, Slopen ME, Pfeiffer MR, McVeigh KH (2012) School-age outcomes of late preterm infants in New York City. Am J Obstet Gynecol 206: 222 e221–226. 22381605 10.1016/j.ajog.2012.01.007

[27] Raju TN, Higgins RD, Stark AR, Leveno KJ (2006) Optimizing care and outcome for late-preterm (near-term) infants: a summary of the workshop sponsored by the National Institute of Child Health and Human Development. Pediatrics 118: 1207–1214. 16951017 10.1542/peds.2006-0018

[28] Yoon BH, Romero R, Park JS, Kim CJ, Kim SH, et al (2000) Fetal exposure to an intra-amniotic inflammation and the development of cerebral palsy at the age of three years. Am J Obstet Gynecol 182: 675–681. 10739529 10.1067/mob.2000.104207

[29] Yoon BH, Romero R, Park JS, Kim M, Oh SY, et al (2000) The relationship among inflammatory lesions of the umbilical cord (funisitis), umbilical cord plasma interleukin 6 concentration, amniotic fluid infection, and neonatal sepsis. Am J Obstet Gynecol 183: 1124–1129. 11084553 10.1067/mob.2000.109035

[30] Rovira N, Alarcon A, Iriondo M, Ibanez M, Poo P, et al (2011) Impact of histological chorioamnionitis, funisitis and clinical chorioamnionitis on neurodevelopmental outcome of preterm infants. Early Hum Dev 87: 253–257. 21354722 10.1016/j.earlhumdev.2011.01.024

[31] Zanardo V, Vedovato S, Suppiej A, Trevisanuto D, Migliore M, et al (2008) Histological inflammatory responses in the placenta and early neonatal brain injury. Pediatr Dev Pathol 11: 350–354. 18275252 10.2350/07-08-0324.1

[32] Polam S, Koons A, Anwar M, Shen-Schwarz S, Hegyi T (2005) Effect of chorioamnionitis on neurodevelopmental outcome in preterm infants. Arch Pediatr Adolesc Med 159: 1032–1035. 16275792 10.1001/archpedi.159.11.1032

[33] Yoon BH, Romero R, Kim CJ, Jun JK, Gomez R, et al (1995) Amniotic fluid interleukin-6: a sensitive test for antenatal diagnosis of acute inflammatory lesions of preterm placenta and prediction of perinatal morbidity. Am J Obstet Gynecol 172: 960–970. 7892891 10.1016/0002-9378(95)90028-4

[34] Mi Lee S, Romero R, Lee KA, Jin Yang H, Joon Oh K, et al (2011) The frequency and risk factors of funisitis and histologic chorioamnionitis in pregnant women at term who delivered after the spontaneous onset of labor. J Matern Fetal Neonatal Med 24: 37–42. 20698737 10.3109/14767058.2010.482622 PMC3163442

[35] Shim SS, Romero R, Hong JS, Park CW, Jun JK, et al (2004) Clinical significance of intra-amniotic inflammation in patients with preterm premature rupture of membranes. Am J Obstet Gynecol 191: 1339–1345. 15507963 10.1016/j.ajog.2004.06.085

[36] Bastek JA, Sammel MD, Rebele EC, Srinivas SK, Elovitz MA (2010) The effects of a preterm labor episode prior to 34 weeks are evident in late preterm outcomes, despite the administration of betamethasone. Am J Obstet Gynecol 203: 140 e141–147. 20522406 10.1016/j.ajog.2010.02.065

[37] ACOG Committee Opinion No. 402: Antenatal corticosteroid therapy for fetal maturation. Obstet Gynecol 111: 805–807. 18310392 10.1097/AOG.0b013e318169f722

[38] Roberts D, Dalziel S (2006) Antenatal corticosteroids for accelerating fetal lung maturation for women at risk of preterm birth. Cochrane Database Syst Rev 3: CD004454. 10.1002/14651858.CD004454.pub2 16856047

[39] Choi SJ, Song SE, Seo ES, Oh SY, Kim JH, et al (2009) The effect of single or multiple courses of antenatal corticosteroid therapy on neonatal respiratory distress syndrome in singleton versus twin pregnancies. Aust N Z J Obstet Gynaecol 49: 173–179. 19432606 10.1111/j.1479-828X.2009.00970.x

[40] Porto AM, Coutinho IC, Correia JB, Amorim MM (2011) Effectiveness of antenatal corticosteroids in reducing respiratory disorders in late preterm infants: randomised clinical trial. BMJ 342: d1696. 21487057 10.1136/bmj.d1696 PMC3075234

[41] Gyamfi-Bannerman C, Gilbert S, Landon MB, Spong CY, Rouse DJ, et al (2012) Effect of antenatal corticosteroids on respiratory morbidity in singletons after late-preterm birth. Obstet Gynecol 119: 555–559. 22353953 10.1097/AOG.0b013e31824758f6 PMC3338333

[42] Ventolini G, Neiger R, Mathews L, Adragna N, Belcastro M (2008) Incidence of respiratory disorders in neonates born between 34 and 36 weeks of gestation following exposure to antenatal corticosteroids between 24 and 34 weeks of gestation. Am J Perinatol 25: 79–83. 18188800 10.1055/s-2007-1022470

[43] Bastek JA, Srinivas SK, Sammel MD, Elovitz MA (2010) Do neonatal outcomes differ depending on the cause of preterm birth? A comparison between spontaneous birth and iatrogenic delivery for preeclampsia. Am J Perinatol 27: 163–169. 19644790 10.1055/s-0029-1234036

[44] Lee J, Seong HS, Kim BJ, Jun JK, Romero R, et al (2009) Evidence to support that spontaneous preterm labor is adaptive in nature: neonatal RDS is more common in “indicated” than in “spontaneous” preterm birth. J Perinat Med 37: 53–58. 19099368 10.1515/JPM.2009.036 PMC2887663

[45] Fernandez-Carrocera LA, Gonzalez-Mora E (2004) [Neurodevelopmental disorders in children with an antecedent of subependymal/intraventricular hemorrhage at 3 years of age]. Gac Med Mex 140: 367–373. 15456146

[46] Khan RL, Nunes ML, Garcias da Silva LF, da Costa JC (2008) Predictive value of sequential electroencephalogram (EEG) in neonates with seizures and its relation to neurological outcome. J Child Neurol 23: 144–150. 18160554 10.1177/0883073807308711

[47] Pillekamp F, Hermann C, Keller T, von Gontard A, Kribs A, et al (2007) Factors influencing apnea and bradycardia of prematurity - implications for neurodevelopment. Neonatology 91: 155–161. 17377399 10.1159/000097446

[48] Shapiro SM (2003) Bilirubin toxicity in the developing nervous system. Pediatr Neurol 29: 410–421. 14684236 10.1016/j.pediatrneurol.2003.09.011

